# Supplementary material for: Comparison of Two Nuclear Magnetic Resonance Spectroscopy Methods for the Measurement of Lipoprotein Particle Concentrations
Source: Biomedicines. 2022 Jul 21;10(7):1766. doi: 10.3390/biomedicines10071766 (PMC9312544; doi:10.3390/biomedicines10071766)

SUPPLEMENTAL MATERIALS

Comparison of two Nuclear Magnetic Resonance Spectroscopy Methods for the Measurement of Lipoprotein Particle Concentrations

Running Title: Comparison of two NMR Methods

Martin Rief, MD[1] (martin.rief@medunigraz.at)
Reinhard Raggam, MD[2] (reinhard.raggam@medunigraz.at)
Peter Rief, MD PhD[2] (peter.rief@medunigraz.at)
Philipp Metnitz, Prof. MD PhD[1] (philipp.metnitz@medunigraz.at)
Tatjana Stojakovic, MD[3] (tatjana.stojakovic@uniklinikum.kages.at)
Markus Reinthaler, MD[4] (markus.reinthaler@charite.de)
Marianne Brodmann, Prof. MD[2] (marianne.brodmann@medunigraz.at)
Winfried März, Prof. MD[3] (winfried.maerz@medunigraz.at)
Hubert Scharnagl, Prof. PhD[5 *] (hubert.scharnagl@medunigraz.at)
Günther Silbernagel, Prof. MD PhD[2] (guenther.silbernagel@medunigraz.at)

[1] Division of General Anaesthesiology, Emergency- and Intensive Care Medicine, Medical University of Graz, Graz, Austria

[2] Division of Angiology, Department of Internal Medicine, Medical University of Graz, Graz, Austria

[3] Clinical Institute of Medical and Chemical Laboratory Diagnostics, University Hospital Graz Graz, Graz, Austria

[4] Charité-Universitätsmedizin Berlin (CBF) Berlin, Germany and Institute of Biomaterial Science, Helmholtz-Zentrum Geesthacht, Teltow, Germany

[5] Clinical Institute of Medical and Chemical Laboratory Diagnostics, Medical University of Graz, Graz, Austria

Supplemental Table S1

Title: Lipofit values under the detection limit

(C= cholesterol. HDL= high density lipoproteins. LHDL= large high density lipoproteins . mg/dl= milligram per deciliter. µmol/l= micromoles per liter. nmol/l= nanomoles per liter. p= particles. SHDL= small high density lipoproteins . SLDL= small low density lipoproteins . TG= triglycerides)

Parameter	***Value***	***Quantity***
SLDL-p	<152 nmol/l	4
LHDL-p	<2780 µmol/l	32
SHDL-p	<6034 µmol/l	2
TG	<44 mg/dl	4
HDL-C	<17 mg/dl	1

Supplemental Table S2

Title: Additional analyses from lipoprofile

([a]p<0.05. [b]p<0.01. [c]p<0.001. apoB= apolipoprotein B. C= cholesterol. HDL= high density lipoproteins. l= liter. LDL= low density lipoproteins. LHDL= large high density lipoproteins . LLDL= large low density lipoproteins. LVLDL= large very low density lipoproteins. MHDL= medium high density lipoproteins. MLDL= medium low density lipoproteins. MVLDL= medium very low density lipoproteins. p= particles. SHDL= small high density lipoproteins . SLDL= small low density lipoproteins . SVLDL= small very low density lipoproteins . TC= total cholesterol. TG= triglycerides. VLDL= very low density lipoproteins. VLVLDL= very large very low density lipoproteins. VSVLDL= small very low density lipoproteins)

Lipoprofile	VLDL-p	VLVLDL-p	LVLDL-p	MVLDL-p	SVLDL-p	VSVLDL-p	LDL-p	LLDL-p	MLDL-p	SLDL-p	HDL-p	HDL-p H1	HDL-p H2	HDL-p H3	HDL-p H4	HDL-p H5	HDL-p H6	HDL-p H7	LHDL-p	MHDL-p	SHDL-p	TC	TG	HDL-C	LDL-C	apoB
VLDL-p	1	0.16	0.47[c]	0.57[c]	0.59[c]	0.69[c]	0.53[c]	0.19[a]	0.42[c]	0.17[a]	-0.02	-0.12	0.13	0.19[a]	-0.18[a]	-0.27[c]	0.09	-0.38[c]	-0.23[b]	0.03	0.03	0.6[c]	0.59[c]	-0.25[b]	0.59[c]	0.6[c]
VLVLDL-p	0.16	1	0.54[c]	0.39[c]	-0.11	0.17[a]	0.05	-0.32[c]	0.07	0.29[c]	0.08	0.22[b]	0.11	-0.17[a]	0.01	-0.06	-0.18[a]	-0.18[a]	-0.21[b]	-0.15	0.21[a]	0.06	0.72[c]	-0.23[b]	-0.09	0.21[a]
LVLDL-p	0.47[c]	0.54[c]	1	0.76[c]	0.04	0.35[c]	0.18[a]	-0.35[c]	0.22[b]	0.32[c]	0.09	0.09	0.24[b]	-0.02	-0.08	-0.22[b]	-0.15	-0.35[c]	-0.35[c]	-0.08	0.23[b]	0.2[a]	0.81[c]	-0.32[c]	0.06	0.25[b]
MVLDL-p	0.57[c]	0.39[c]	0.76[c]	1	0.31[c]	0.21[b]	0.29[c]	-0.29[c]	0.34[c]	0.29[c]	0.22	0.26[c]	0.3[c]	-0.12	-0.02	-0.15	-0.15	-0.37[c]	-0.32[c]	-0.14	0.38[c]	0.34[c]	0.76[c]	-0.22[b]	0.21[b]	0.35[c]
SVLDL-p	0.59[c]	-0.11[a]	0.04	0.31[c]	1	-0.16	0.6[c]	0.45[c]	0.46[c]	-0.01	0.21[a]	0.03	0.28[c]	0.13	-0.14	-0.11	0.14	-0.3[c]	-0.08	0.01	0.23[b]	0.67[c]	0.22[b]	0.01	0.71[c]	0.58[c]
VSVLDL-p	0.69[c]	0.17[a]	0.65[c]	0.21[b]	-0.16	1	0.12	-0.04	0.08	0.14	-0.28[c]	-0.27[c]	-0.15	0.18[a]	-0.12	-0.22[b]	0.05	-0.15	-0.15	0.08	-0.27[c]	0.14	0.37[c]	-0.26[c]	0.12	0.2
LDL-p	0.53[c]	0.05	0.18[a]	0.29[c]	0.6[c]	0.12	1	0.42[c]	0.75[c]	0.31[c]	0.3[c]	0.12	0.31[c]	0.15	-0.03	-0.01	-0.02	-0.28[c]	-0.12	0.12	0.3[c]	0.9[c]	0.35[c]	0.07	0.92[c]	0.95[c]
LLDL-p	0.19[a]	0.32[c]	0.35[c]	-0.29[c]	0.45[c]	-0.04	0.42[c]	1	0.09	-0.32[c]	0.01	-0.17[a]	-0.1	0.19[a]	0.06	0.05	0.32[c]	0.15	0.3[c]	0.23[b]	-0.17[a]	0.52[c]	-0.28[c]	0.32[c]	0.61[c]	0.4[c]
MLDL-p	0.42[c]	0.07	0.22[b]	0.34[c]	0.46[c]	0.08	0.75[c]	0.09	1	-0.15	0.41[c]	0.17[a]	0.49[c]	-0.04	0.08	0.02	-0.17[a]	-0.22[b]	-0.18[a]	0.03	0.46[c]	0.71[c]	0.33[c]	0.12	0.68[c]	0.7[c]
SLDL-p	0.17[a]	0.29[c]	0.32[c]	0.29[c]	-0.01	0.14	0.31[c]	-0.32[c]	-0.15	1	-0.04	0.14	-0.04	0.12	-0.21[b]	-0.09	-0.1	-0.33[c]	-0.23[b]	-0.05	0.05	0.09	0.39[c]	-0.32[c]	0.08	0.3[c]
HDL-p	-0.02	0.08	0.09	0.22[b]	0.21[a]	-0.28[c]	0.3[c]	0.01	0.41[c]	-0.04	1	0.6[c]	0.71[c]	-0.1	0.38[c]	0.33[c]	-0.02	0.01	0.18[a]	0.21[b]	0.88[c]	0.48[c]	0.07	0.64[c]	0.32[c]	0.26[b]
HDL-p H1	-0.12	0.22[b]	0.09	0.26[c]	0.03	-0.27[c]	0.12	-0.17[a]	0.17[a]	0.14	0.6[c]	1	0.13	-0.4[c]	0.16[a]	0.45[c]	-0.16	0.06	0.18[a]	-0.26[c]	0.66[c]	0.2[a]	0.15	0.28[c]	0.1	0.18[a]
HDL-p H2	0.13	0.11	0.24[b]	0.3[c]	0.28[c]	-0.15	0.3[c]	-0.1	0.49[c]	-0.04	0.71[c]	0.13	1	-0.19[a]	0.17[a]	-0.08	-0.2[b]	-0.3[c]	-0.29[c]	-0.05	0.83[c]	0.36[c]	0.21[b]	0.16[a]	0.29[c]	0.24[b]
HDL-p H3	0.19[a]	-0.17[a]	-0.02	-0.12	0.13	0.18[a]	0.15	0.19[a]	-0.04	0.12	-0.1	-0.4[c]	-0.19[a]	1	-0.37[c]	-0.11	0.24[b]	-0.22[b]	0.02	0.67[c]	-0.37[c]	0.1	-0.1	-0.02	0.16[a]	0.07
HDL-p H4	-0.18[a]	0.01	-0.08	-0.02	-0.14	-0.12	-0.029	0.06	0.08	-0.21[b]	0.38[c]	0.16[a]	0.17[a]	-0.37[c]	1	0.02	-0.18[a]	0.22[b]	-0.03	0.45[c]	0.22[b]	0.09	-0.08	0.47[c]	-0.03	-0.04
HDL-p H5	-0.27[c]	-0.06	-0.22[b]	-0.15	-0.11	-0.22[b]	-0.01	0.05	0.02	-0.9	0.33[c]	0.45[c]	-0.08	-0.11	0.02	1	-0.18[a]	0.37[c]	0.6[c]	-0.09	0.19[a]	0.09	-0.19[a]	0.48[c]	0.01	-0.01
HDL-p H6	0.09	-0.18[a]	-0.15	-0.15	0.14	0.05	-0.02	0.32[c]	-0.17[a]	-0.1	-0.02	-0.16	-0.2[a]	0.24[b]	-0.18[a]	-0.18[a]	1	0.15	0.61[c]	0.09	-0.24[b]	0.12	-0.15	0.37[c]	0.06	-0.06
HDL-p H7	-0.38[c]	-0.18[a]	-0.35[c]	-0.37[c]	-0.3[c]	-0.15	-0.28[c]	0.15	-0.22[b]	-0.33[c]	0.01	0.06	-0.3[c]	-0.22[b]	0.22[b]	0.37[c]	0.15	1	0.67[c]	-0.03	-0.19[a]	-0.63	-0.43[c]	0.56[c]	-0.12	-0.2[a]
LHDL-p	0.19[a]	0.21[b]	0.35[c]	-0.32[c]	-0.08	-0.15	-0.12	0.3[c]	-0.18[a]	-0.23[†]	0.18[a]	0.66[c]	-0.29[c]	0.02	-0.03	0.6[c]	0.61[c]	0.67[c]	1	-0.01	-0.12	0.11	-0.36[c]	0.73[c]	0.01	-0.12
MHDL-p	0.42[c]	-0.15	-0.08	-0.14[a]	0.01	0.08	0.12	0.26[b]	0.03	-0.05	0.21[b]	-0.26[c]	-0.05	0.67[c]	0.45[c]	-0.09	0.09	-0.03	-0.01	1	-0.18[a]	0.17[a]	-0.16	0.36[c]	0.13	0.04
SHDL-p	0.17[a]	0.21[a]	0.23[b]	0.38[c]	0.23[b]	-0.27[c]	0.3[c]	-0.17[a]	0.46[c]	0.05	0.88[c]	0.18[a]	0.83[c]	-0.37[c]	0.22[b]	0.19[a]	-0.24[b]	-0.19[a]	-0.12	-0.18[a]	1	0.38[c]	0.25[b]	0.28[c]	0.28[c]	0.28[c]
TC	0.6[c]	0.06	0.2[a]	0.34[c]	0.67[c]	0.14	0.89[c]	0.52[c]	0.71[c]	0.09	0.48[c]	0.2[a]	0.36[c]	0.1	0.09	0.09	0.12	-0.63	0.11	0.17[a]	0.38[c]	1	0.35[c]	0.34[c]	0.95[c]	0.91[c]
TG	0.59[c]	0.72[c]	0.81[c]	0.76[c]	0.22[b]	0.37	0.35[c]	-0.28[c]	0.33[c]	0.39[c]	0.07	0.15	0.21[b]	-0.1	-0.08	-0.19[a]	-0.15	-0.43[c]	-0.36[c]	-0.16	0.25[b]	0.35[c]	1	-0.35[c]	0.19[a]	0.5[c]
HDL-C	-0.25[b]	0.23[b]	-0.32[c]	-0.22[b]	0.01	-0.26[c]	0.07	0.32[c]	0.12	-0.32[c]	0.64[c]	0.28[c]	0.16[a]	-0.02	0.47[c]	0.48[c]	0.37[c]	0.56[c]	0.73[c]	0.36[c]	0.28[c]	0.34[c]	-0.35[c]	1	0.18[a]	0.03
LDL-C	0.59[c]	-0.09	0.06	0.21[b]	0.71[c]	0.12	0.92[c]	0.61[c]	0.68[c]	0.08	0.32[c]	0.1	0.29[c]	0.16[a]	-0.03	0.01	0.06	-0.12	0.01	0.13	0.28[c]	0.95[c]	0.19[a]	0.18[a]	1	0.89[c]
apoB	0.6[c]	0.21[a]	0.25[b]	0.35[c]	0.58[c]	0.2[a]	0.95[c]	0.4[c]	0.7[c]	0.3[c]	0.26[b]	0.18[a]	0.24[b]	0.07	-0.04	-0.01	-0.06	-0.2[a]	-0.12	0.04	0.25[c]	0.91[c]	0.5[c]	0.03	0.89[c]	1

Supplemental Table S3

Title: Compared parameters and units of the corresponding parameters

(C= cholesterol. HDL= high density lipoproteins. l= liter. LDL= low density lipoproteins. mg/dl= milligram per deciliter. µmol/l= micromoles per liter. nmol/l= nanomoles per liter. NMR= nuclear magnetic resonance spectroscopy. p= particles. TC= total cholesterol. TG= triglycerides. VLDL= very low density lipoproteins)

Lipoprofile (NMR)	*Lipofit (NMR)*	*Standard method (Ultracentrifugation)*	*Unit*
TC	TC	TC	mg/dl
TG	TG	TG	mg/dl
HDL-C	HDL-C	HDL-C	mg/dl
LDL-C	LDL-C	LDL-C	mg/dl
Large + very large VLDL	Large VLDL	No value	nmol/l
LDL-p	LDL-p	No value	nmol/l
Small + medium LDL-p	Small LDL-p	No value	nmol/l
Large LDL-p	Large LDL-p	No value	nmol/l
HDL-p	HDL-p	No value	µmol/l
HDL-p (H4-H7)	Large HDL-p	No value	µmol/l
HDL-p (H1-H3)	Small HDL-p	No value	µmol/l

Supplemental Table S4

Title: NMR values compared due to their size

(HDL= high density lipoproteins. l= liter. LDL= low density lipoproteins. µmol/l= micromoles per liter. *nmol/l= nanomoles per liter. p= particles. VLDL= very low density lipoproteins)*

Lipoprofile (Sub)Class	*Lipoprofile size (nm)*	*Lipofit (Sub)Class*	*Lipofit size (nm)*
Total VLDL-p	24-240		
Very large VLDL-p	90-240	***Large VLDL-p***	60-200
Large VLDL-p	50-89		
Medium VLDL-p	37-49		
Small VLDL-p	30-36		
Very small VLDL-p	24-29		
Total LDL-p	19-23	***Total LDL-p***	18-23
Large LDL-p	21.5-23	***Large LDL-p***	21.2-23
Medium LDL-p	20.5-21.4	***Small LDL-p***	18-21.2
Small LDL-p	19-20.4		
Total HDL-p	7.4-13	***Total HDL-p***	7.3-13
Large HDL-p	9.6-13	***Large HDL-p***	8.8-13
H7-p	12		
H6-p	10.8		
H5-p	10.3		
H4-p	9.5		
Medium HDL-p	8.1-9.5	***Small HDL-p***	7.3-8.8
H3-p	8.7		
Small HDL-p	7.4-8		
H2-p	7.8		
H1-p	7.4		

Supplemental Table S5

Title: Comparison of standard lipids between ß-quantification and the lipofit and lipoprofile methods

(C= cholesterol. CI= confidence interval. HDL= high density lipoproteins. MD= mean difference. LDL= low density lipoproteins. LoA= limit of agreement. TC= total cholesterol. TG= triglycerides)

Parameter	***Lipoprofile***			***Lipofit***		
	r-correlation	***Passing Bablok***	***Bland Altman***	***r- correlation***	***Passing Bablok***	***Bland Altman***
		Slope (95%CI)	***MD (95%CI)***		***Slope (95%CI)***	***MD (95%CI)***
TC	0.964	0.889 (0.861 – 0.921)	- 8.2 (-10.5 – -5.91)	0.947	0.914 (0.864 – 0.963)	-4.56 (-7.21 – -1.91)
TG	0.979	1.027 (1.000 – 1.045)	2.58 (-0.184 – 5.34)	0.961	0.983 (0.924 – 1.046)	3.19 (-0.571 – 6.94)
LDL-C	0.941	0.960 (0.921 – 1.000)	-4.65 (-6.76 – -2.55)	0.935	0.973 (0.909 – 1.034)	-3.25 (-5.47 – -1.03)
HDL-C	0.837	0.830 (0.756 – 0.897)	-0.593 (-2.18 – 0.991)	0.805	0.786 (0.700 – 0.885)	2.79 (1.07 – 4.5)

Supplemental Table S6

Title: Comparison between the lipofit and lipoprofile methods

([a] compared with LabCorp large + very large VLDL-p. [b] compared with LabCorp small + medium LDL-p. [c] compared with LabCorp H4-H7 HDL-p. [d] compared with LabCorp H1-H3 HDL-p. C= cholesterol. HDL= high density lipoproteins. LDL= low density lipoproteins. LHDL= large high density lipoproteins. LLDL= large low density lipoproteins. LVLDL= large very low density lipoproteins. MD= mean difference. p= *particles. SHDL= small high density lipoproteins. SLDL= small low density lipoproteins. TC= total cholesterol. TG= triglycerides)*

Parameter	***r-correlation***	***Passing Bablok regression***		***Bland Altman analysis***	
		slope	***95% CI***	***MD***	***95% CI***
TC	0.950	1.026	0.966 – 1.082	3.6	1.3 – 5.94
TG	0.964	0.988	0.932 – 1.050	0.6	-2.87 – 4.08
LDL-C	0.953	1.000	0.946 – 1.067	1.4	-0.46 – 3.26
HDL-C	0.921	0.958	0.889 – 1.000	3.4	2.55 – 4.21
LVLDL-p[a]	0.898	0.980	0.865 – 1.157	1.52	1.1 – 1.94
LDL-p	0.908	1.057	0.984 – 1.138	-153.8	-185 – -122
LLDL-p	0.607	1.272	1.077 – 1.468	299.6	261 – 338
SLDL-p[b]	0.789	0.593	0.519 – 0.668	-431	-472 – -389
LDL-size	0.677	0.860	0.738–1.002	-0.001	-0.05–0.048
HDL-p	0.934	1.637	1.532 – 1.770	14.8	14.22 – 15.3
LHDL-p[c]	0.869	1.722	1.540 – 1.903	1.72	1.41 – 2.03
SHDL-p[d]	0.735	1.817	1.642 – 2.058	12.8	11.9 – 13.7
HDL-size	0.843	1.014	0.916–1.099	-0.13	-0.175–-0.086

Supplemental Figure S1

Title: Comparison of standard lipids between ß-quantification and NMR (Bland Altman plots)

(dashed line = limits of agreement, continuous horizontal line = mean difference; C= cholesterol. HDL= high density lipoproteins. LDL= low density lipoproteins. TC= total cholesterol. TG= triglycerides)

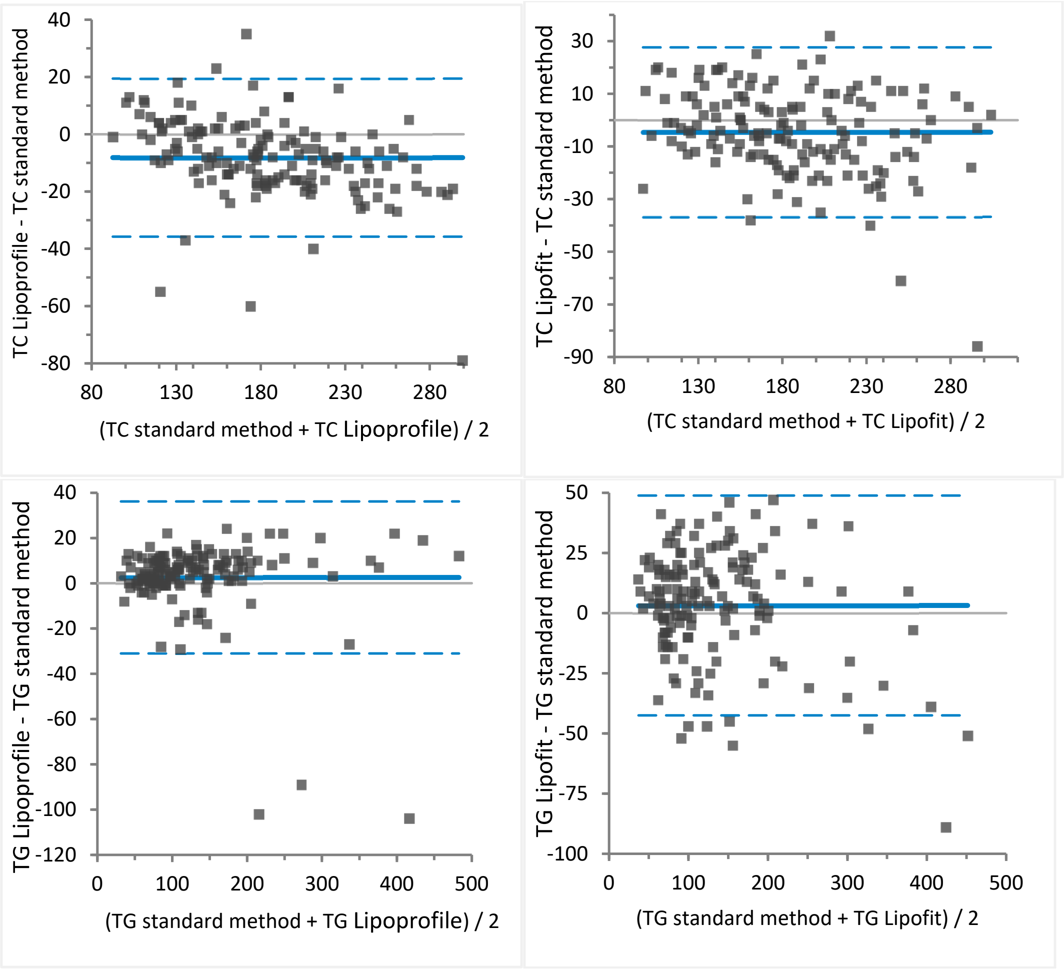

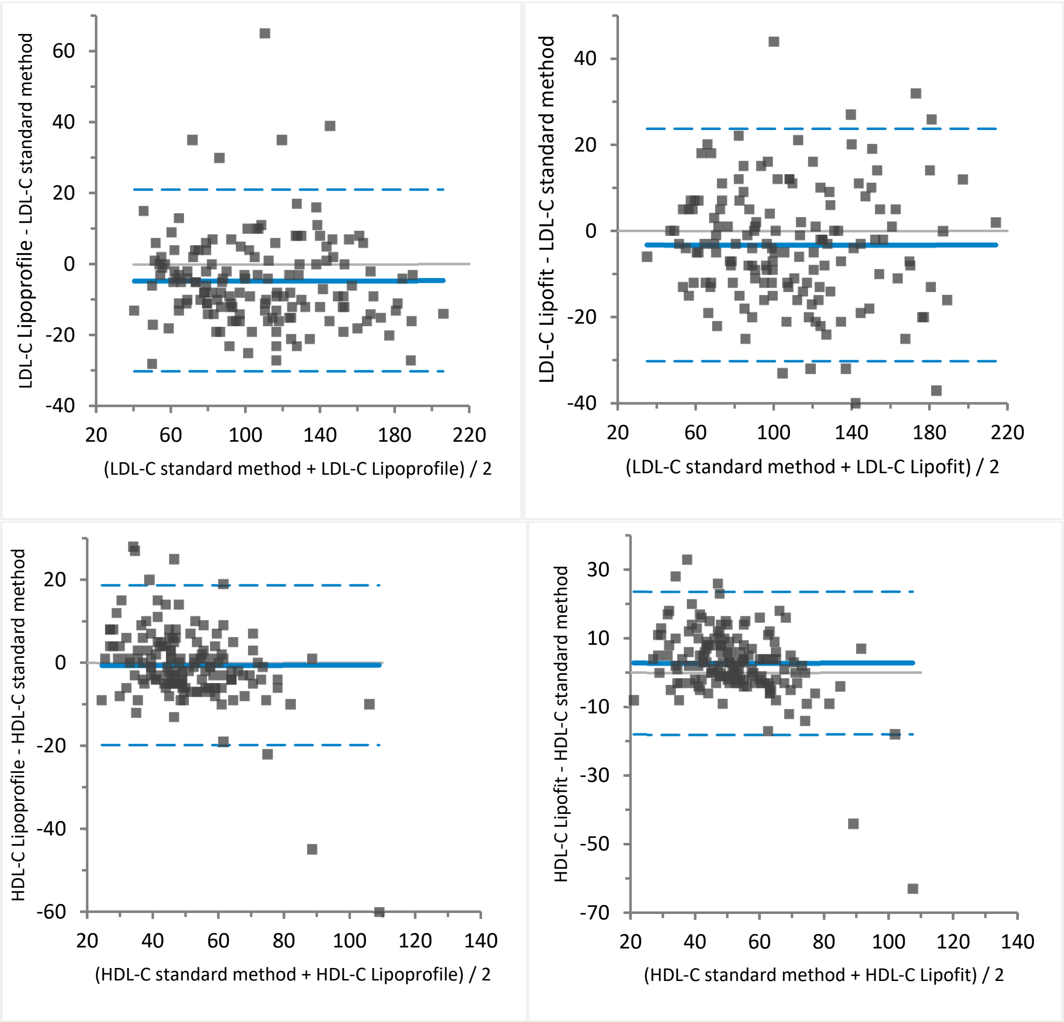

LDL-C Lipoprofile - LDL-C standard method
(LDL-C standard method + LDL-C Lipoprofile) / 2
LDL-C Lipofit - LDL-C standard method
(LDL-C standard method + LDL-C Lipofit) / 2
HDL-C Lipoprofile - HDL-C standard method
(HDL-C standard method + HDL-C Lipoprofile) / 2
HDL-C Lipofit - HDL-C standard method
(HDL-C standard method + HDL-C Lipofit) / 2

Supplemental Figure S2

Title: Comparison of lipoprotein particles between the lipoprofile and lipofit methods (Bland Altman plots)

(dashed line = limits of agreement, continuous horizontal line = mean difference; HDL= high density lipoproteins. LDL= low density lipoproteins. p= particles. VLDL= very low density lipoproteins)

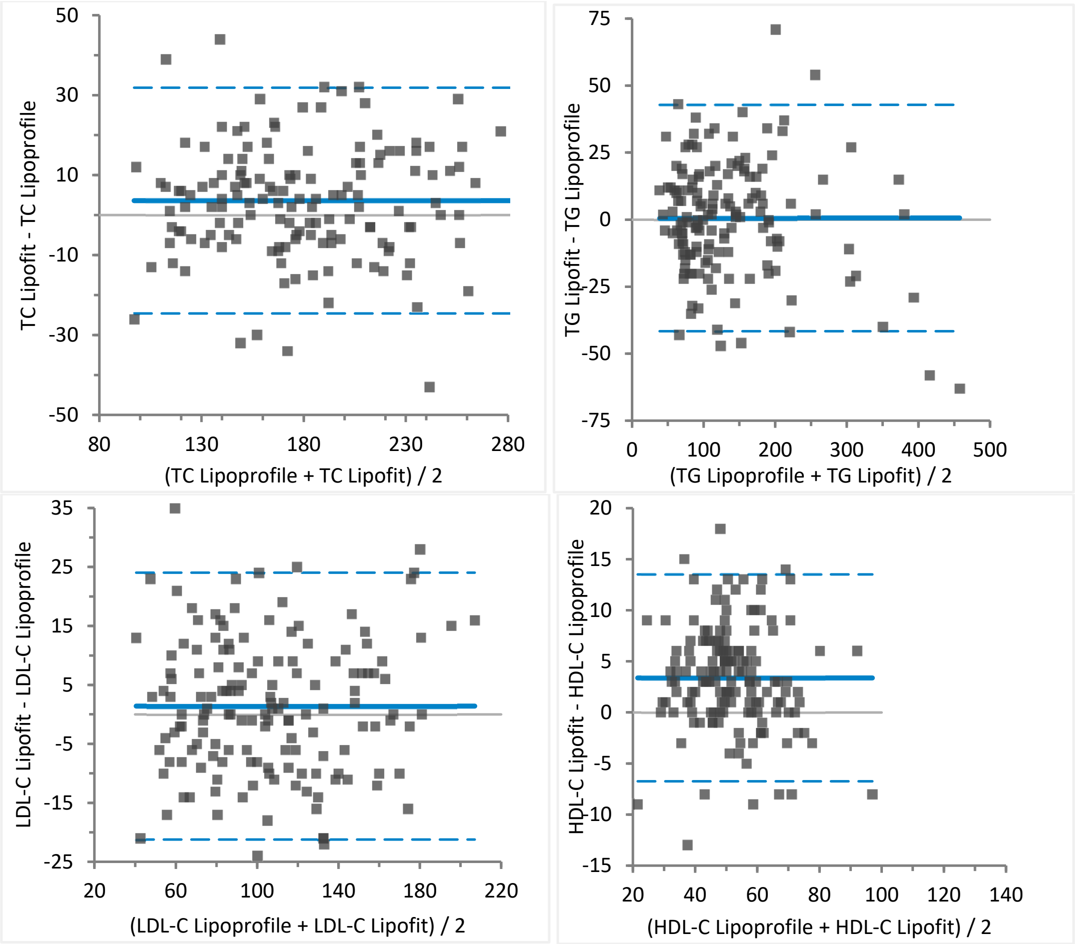

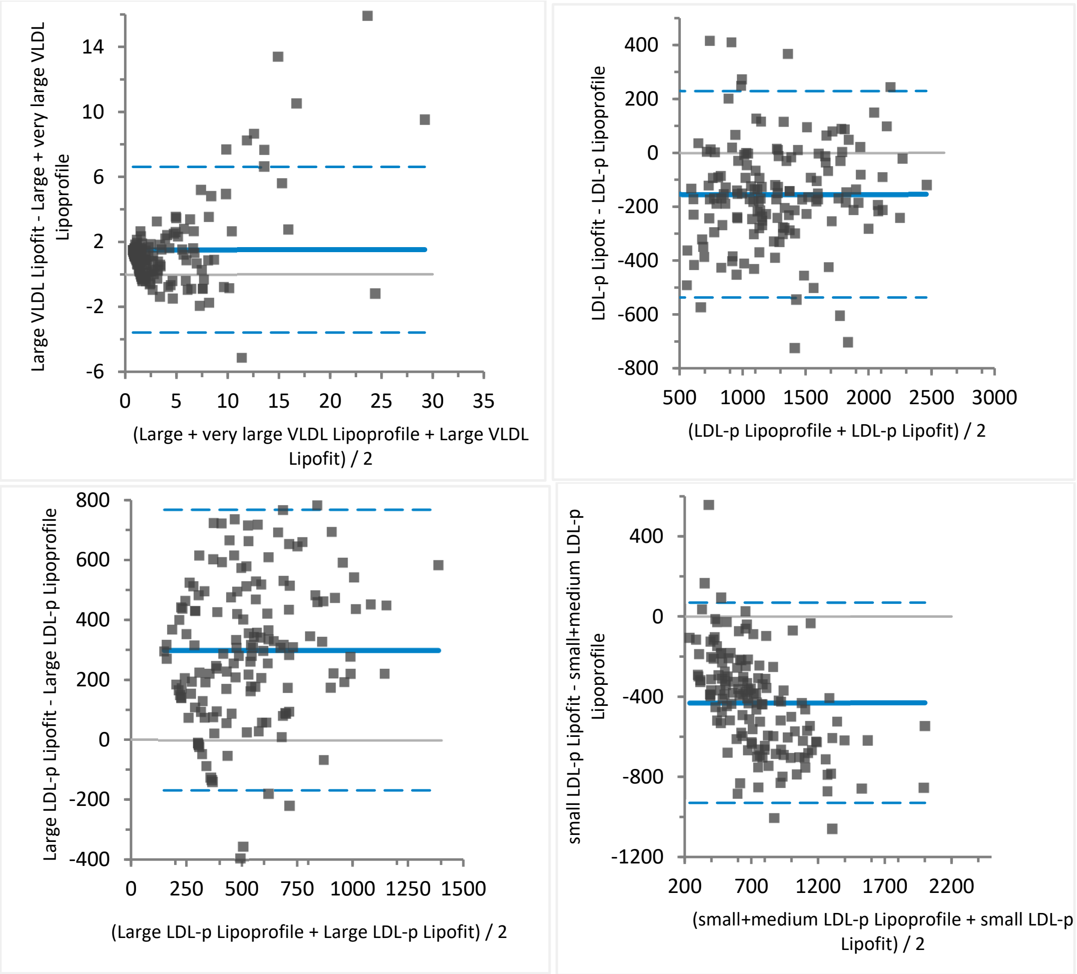
Large VLDL Lipofit - Large + very large VLDL Lipoprofile
14
10
6
2
-2
-6
0
5
10
15
20
25
30
35
(Large + very large VLDL Lipoprofile + Large VLDL Lipofit) / 2
LDL-p Lipofit - LDL-p Lipoprofile
400
200
0
-200
-400
-600
-800
500
1000
1500
2000
2500
3000
(LDL-p Lipoprofile + LDL-p Lipofit) / 2
Large LDL-p Lipofit - Large LDL-p Lipoprofile
800
600
400
200
0
-200
-400
0
250
500
750
1000
1250
1500
(Large LDL-p Lipoprofile + Large LDL-p Lipofit) / 2
small LDL-p Lipofit - small+medium LDL-p Lipoprofile
400
0
-400
-800
-1200
200
700
1200
1700
2200
(small+medium LDL-p Lipoprofile + small LDL-p Lipofit) / 2

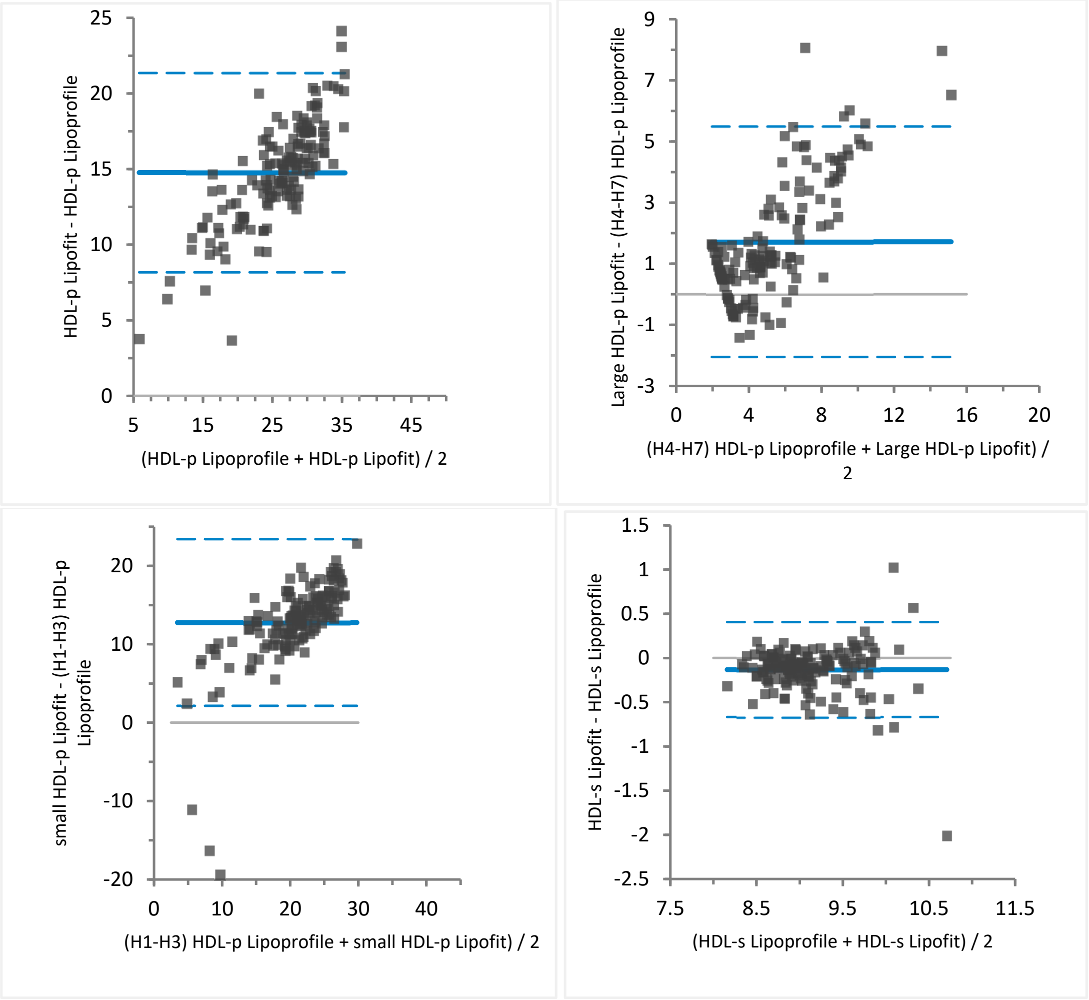

HDL-p Lipofit - HDL-p Lipoprofile
25
20
15
10
5
0
5
15
25
35
45
(HDL-p Lipoprofile + HDL-p Lipofit) / 2
Large HDL-p Lipofit - (H4-H7) HDL-p Lipoprofile
9
7
5
3
1
-1
-3
0
4
8
12
16
20
(H4-H7) HDL-p Lipoprofile + Large HDL-p Lipofit) / 2
small HDL-p Lipofit - (H1-H3) HDL-p Lipoprofile
20
10
0
-10
-20
0
10
20
30
40
(H1-H3) HDL-p Lipoprofile + small HDL-p Lipofit) / 2
HDL-s Lipofit - HDL-s Lipoprofile
1.5
1
0.5
0
-0.5
-1
-1.5
-2
-2.5
7.5
8.5
9.5
10.5
11.5
(HDL-s Lipoprofile + HDL-s Lipofit) / 2

Supplemental Figure S3

Title: Comparison of standard lipids between the lipoprofile and lipofit methods

The figures show the Passing-Bablok regression for total cholesterol (top left), triglycerides (top right), low density lipoprotein cholesterol (bottom left, and high density lipoprotein cholesterol (bottom right). The respective slopes of the regression lines (red) were 1.026 (TC), 0.988 (TG), 1.000 (LDL-C), and 0.958 (HDL-C), respectively. The grey line represents the line of identity.

(C= cholesterol. HDL= high density lipoproteins. LDL= low density lipoproteins. TC= total cholesterol. TG= triglycerides)

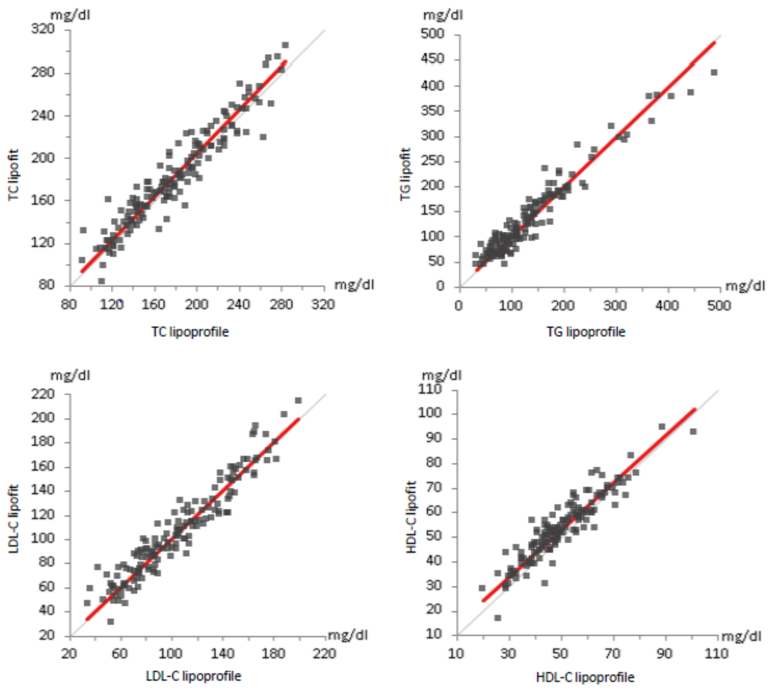

Supplement: Supplementary file 1 [file biomedicines-10-01766-s001.zip › biomedicines-1747155-supplementary.pdf]
